# Synergistic Action of Sodium Selenite with some Antidepressants and Diazepam in Mice

**DOI:** 10.3390/pharmaceutics10040270

**Published:** 2018-12-12

**Authors:** Ewa Kędzierska, Lila Dąbkowska, Paweł Obierzyński, Magdalena Polakowska, Ewa Poleszak, Piotr Wlaź, Katarzyna Szewczyk, Jolanta Kotlińska

**Affiliations:** 1 Department of Pharmacology and Pharmacodynamics, Faculty of Pharmacy with Division of Medical Analytics, Medical University of Lublin, Chodźki 4a, 20-093 Lublin, Poland; liladab93@gmail.com (L.D.); jolanta.kotlinska@umlub.pl (J.K.); 2 Human Anatomy Research Group, Department of Human Anatomy, Medical University of Lublin, Jaczewskiego 4, 20-400 Lublin, Poland; pawel.obierzynski@interia.pl; 3 Institute of Biochemistry and Biophysics Polish Academy of Sciences, Pawińskiego 5A, 02-106 Warsaw, Poland; polakowska.magda@gmail.com; 4 Department of Applied Pharmacy, Medical University of Lublin, Chodźki 1, 20-093 Lublin, Poland; ewa.poleszak@umlub.pl; 5 Department of Animal Physiology, Faculty of Biology and Biotechnology, Maria Curie-Skłodowska University, Akademicka 19, 20-033 Lublin, Poland; piotr.wlaz@poczta.umcs.lublin.pl; 6 Department of Pharmaceutical Botany, Medical University of Lublin, Chodźki 1, 20-093 Lublin, Poland; k.szewczyk@umlub.pl

**Keywords:** sodium selenite, forced swim test, elevated plus-maze test, antidepressants, diazepam, mice

## Abstract

Background: The antidepressant and anxiolytic effects of selenium (Se) have been proven in many studies. This work was aimed at confirming these activities of its inorganic form—sodium selenite—and examining the possible synergy of action with antidepressants and diazepam. Methods: The antidepressant- and anxiolytic-like activity of Se was assessed using forced swim tests (FSTs) and elevated plus-maze test (EPMs). Spontaneous locomotor activity was measured using photoresistor actimeters. The experiments were conducted on male Albino Swiss mice. Results: Sodium selenite (0.5 mg/kg) reduced the immobility time in the FSTs and extended time spent in the open arms of EPMs without affecting locomotor activity The combined administration of Se at an ineffective dose (0.25 mg/kg) together with imipramine (15 mg/kg), fluoxetine (5 mg/kg), tianeptine (10 mg/kg), but not with reboxetine (2.5 mg/kg), resulted in a reduction of immobility time in FSTs, and with a threshold dose of diazepam (0.25 mg/kg) led to the prolongation of time spent in the open arms of the EPM. Moreover, the antidepressant-like effect of Se (0.5 mg/kg) was significantly reduced by pretreatment with p-chlorophenylalanine (100 mg/kg). Conclusions: The results may indicate the participation of serotonergic transmission to antidepressant action of Se and GABA-ergic transmission to its anxiolytic effects.

## 1. Introduction

Depression is a common mental illness that leads to mental impairment, physical disability, and socioeconomic burden [1]. It is one of the most widespread psychiatric disorder in the world, estimated to affect up to 21% of the population, and according to World Health Organization (WHO) predictions, it will be the second most frequently occurring disease in 2020 [2]. It affects a patient’s daily life, family, social, and professional relationships. Almost a million people die each year due to suicide, and 20 times more try to commit it [3]. 

Depression changes the physiology and chemistry of the central and peripheral nervous systems. Therefore, it is important to consider depression not only as a psychological disorder but also as a systemic illness that affects the entire body [4]. Current hypotheses regarding the etiology of the depressive disorder tend to integrate monoaminergic, neuroendocrine, and immunological concepts with those based on oxidative stress, neuronal plasticity, and neurogenesis disturbances (the role of brain derived neurotrophic factor, BDNF). Many research papers highlight the involvement of several pathways i.e., serum lipids, hypothalamic–pituitary–adrenal axis (HPA axis), inflammatory system, gut microbiota, kynurenine pathway [5,6,7,8,9,10,11,12,13]. This complexity of various factor interactions may be the cause of increased risk of atherosclerosis and cardiovascular changes such as ischemic heart disease [14] and stroke [15] as well as endocrine dysregulation [16]. In addition, such mental disorder may be associated with the development of tumors [17]. 

An important feature of depressive disorders is also persistent anxiety, the so-called “free-floating anxiety”. The diagnosis of anxiety is undoubtedly important in the treatment of depression, because of less favorable prognosis in people with depression complicated by anxiety than with depression itself [18].

Selenium (Se) is a trace element, currently recognized as an indispensable micronutrient for animal and human nourishment; however, it is toxic in larger quantities [19]. It is an important component of numerous metabolic pathways, including thyroid hormone metabolism, due to the fact that it is included in the active sites of a wide range of proteins as selenocysteine. The most important include: glutathione peroxidase, thioredoxin reductase, selenoprotein P, and tetraiodothyronine deiodinase. Selenium protects intracellular structures against oxidative damage and has immune functions [20,21,22]. The role of Se in the central nervous system (CNS) is mainly demonstrated by the fact that in the case of deficiency of this microelement, it is first delivered to the brain at the expense of other tissues. Its deficit causes irreversible changes and impairs both cognitive and motor functions; it can lead to progressive cerebellar atrophy [23]. The very important functions of Se is due to its antioxidant activity. Production of reactive oxygen species may contribute to numerous neuronal and neuromuscular disorders, including Alzheimer’s and Parkinson’s disease, amyotrophic lateral sclerosis or Duchenne muscular dystrophy [24]. Protection of the hippocampus, the substantia nigra, and frontal cortex against oxidative damage is possible primarily due to the activities of the abovementioned selenoproteins. However, Se may also act directly on the CNS in the form of selenite or other as yet unexplained ways. Its neuroprotective properties can be attributed to the ability to modulate Ca^2+^ channels [25] or to anti-inflammatory and immunostimulatory properties [20,26]. Literature data indicates that Se may be involved in the mechanisms of neurotransmission affecting the dopaminergic system [27], cholinergic, glutamatergic pathways or those associated with γ-aminobutyric acid (GABA) [22]. Animals with lower levels of Se in the brain may have spastic conditions, impaired mobility, and spontaneous seizures. A few preclinical and clinical studies showed the positive role of this microelement in the improvement of cognitive functions, while studies on various animal species have demonstrated the effectiveness of sodium selenite in epilepsy models. In addition, Se deficiency in humans was associated with depressive states, and its supplementation led to improved mood and reduced anxiety [28,29]. In summary, the aforementioned research suggests that Se is a strong neuroprotective agent in the CNS and may be potentially helpful in the prevention and treatment of neuropsychiatric disorders; however, its mechanism of action is not well understood and requires further research. 

Despite the huge development in medicine over the last years, treatment of some diseases is still impossible, insufficiently effective or involves the occurrence of numerous adverse reactions. For this reason, it is extremely important to develop novel treatment regimens, as well as search for new drugs or other clinical applications for drugs already known. 

Numerous studies on animals have demonstrated the antidepressant- and anxiolytic-like activity of organic Se compounds, while data on such activities for its inorganic forms are lacking [22,28,29,30]. Based on the previous results confirming anxiolytic and antidepressant-like activity of sodium selenite [31], in this work an attempt was made to assess the mechanisms underlying this action. Due to the coexistence of depression and anxiety in clinical practice and the fact that depression complicated by anxiety is more difficult to treat, both the antidepressant- and anxiolytic-like effects of sodium selenite were studied. This work was aimed at investigating the possible synergy of action resulting from the combined administration of Se with antidepressants from various classes and anxiolytic diazepam. Additionally, the participation of the serotonin system was examined by using p-chlorophenylalanine (pCPA, serotonin depletor). In experiments, the forced swim test (FST) and the elevated plus-maze test (EPM) were used, as well as the effect of sodium selenite on the locomotor activity in mice was measured.

## 2. Results

### 2.1. Effect of Se Administration in the FST and on Spontaneous Locomotor Activity

**Figure 1:**
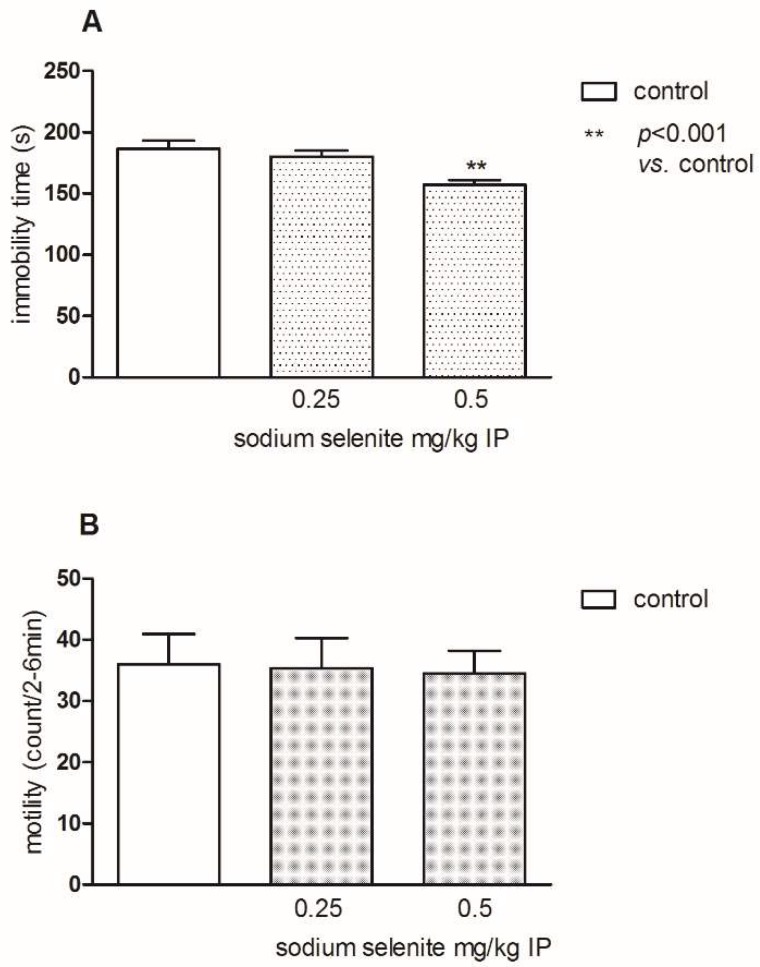
The effect of sodium selenite (at the doses 0.25 and 0.5 mg/kg) on the total duration of immobility in the forced swim test (FST) in mice (**A**) and on the spontaneous locomotor activity in mice (**B**). The values represent the mean of immobility time ± SEM (standard error of the mean) in the FST and the movement of mice between the 2nd and 6th min ± SEM in the locomotor activity test. Sodium selenite was injected intraperitoneally (IP) 30 min before the test. ** *p* < 0.01 vs. control vehicle-treated group (Bonferroni’s test).

Mice that were administered Se at doses of 0.25 and 0.5 mg/kg IP (intraperitoneally) were tested in the FST (Figure 1A) and in spontaneous locomotor activity test (Figure 1B). Selenium significantly reduced the immobility time in FST at the dose 0.5 mg/kg compared to the control group (*p* < 0.01), and was not active at the dose of 0.25 mg/kg (Figure 1A; one-way ANOVA: F(2,26) = 8.259; *p* < 0.01; Bonferroni’s post-hoc test). The administration of Se at the same doses did not change the spontaneous locomotor activity in mice (Figure 1B; one-way ANOVA: F(2,26) = 0.02785; *p* = 0.9726). Considering this result, the ineffective dose 0.25 mg/kg of Se was chosen for further interaction studies with antidepressants in the FST.

### 2.2. Effect of the Administration of Se and Imipramine (IMI) in the FST

**Figure 2:**
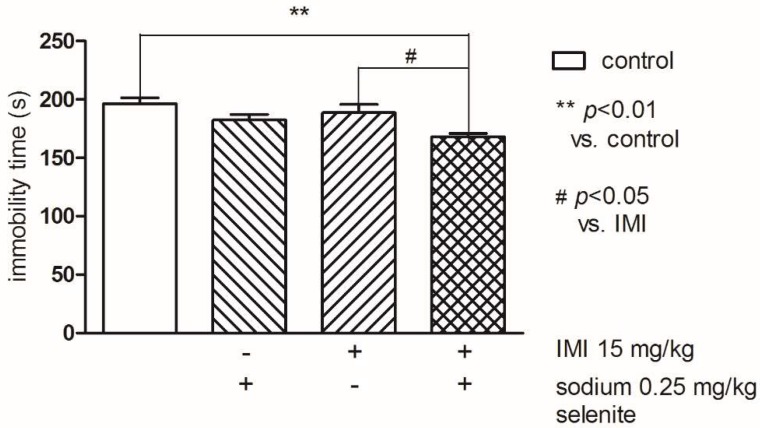
The effects of combined administration of Se and imipramine (IMI) on the immobility time in the FST in mice. The values represent the mean ± SEM. Sodium selenite was injected IP 30 min before the test. ** *p* < 0.01 vs. control vehicle-treated group, # *p* < 0.05 vs. IMI (Bonferroni’s test).

Two-way ANOVA indicated statistically significant differences between groups (control and sodium selenite) (F(1,33) = 9.11; *p* < 0.01). The post-hoc Bonferroni’s test showed that both Se (0.25 mg/kg) and IMI at the threshold dose 15 mg/kg administered alone had no effect on the immobility time. Whereas concomitant treatment of Se and IMI at the abovementioned doses resulted in a statistically significant reduction of the immobility time compared to the control (*p* < 0.01), as well as to IMI (*p* < 0.05) groups (Figure 2).

### 2.3. Effect of the administration of Se and fluoxetine (FLX) in the FST 

**Figure 3:**
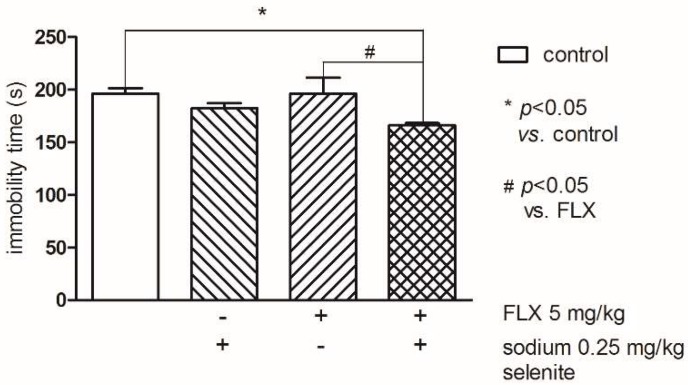
The effects of combined administration of Se and fluoxetine (FLX) on the total duration of immobility in the FST in mice. The values represent the mean ± SEM. Sodium selenite was injected IP 30 min before the test. * *p* < 0.05 vs. control vehicle-treated group, # *p* < 0.05 vs. FLX (Bonferroni’s test).

Two-way ANOVA followed by Bonferroni’s test (groups (control and sodium selenite) F(1,30) = 9.08; *p* < 0.01) indicated that Se (0.25 mg/kg) and FLX at the threshold dose 5 mg/kg administered alone had no effect on the immobility time. Whereas concomitant treatment of Se and FLX at the abovementioned doses resulted in a statistically significant reduction of the immobility time compared to the control (*p* < 0.05) as well as to FLX (*p* < 0.05) groups (Figure 3).

### 2.4. Effect of the Administration of Se and Reboxetine (RB) in the FST

**Figure 4:**
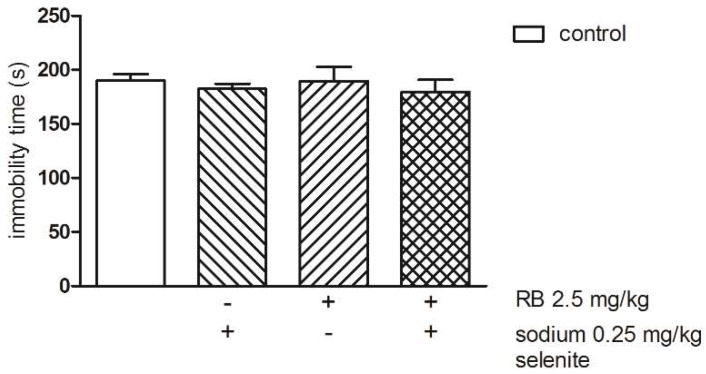
The effects of combined administration of Se and reboxetine (RB) on the total duration of immobility in the FST in mice. The values represent the mean ± SEM. Sodium selenite was injected IP 30 min before the test.

The results depicted in Figure 4 show that both Se at the dose 0.25 mg/kg and RB at the threshold dose 2.5 mg/kg administered alone as well as after concomitant treatment had no effect on the immobility time in mice (two-way ANOVA: groups (control and sodium selenite) F(1,35) = 3.86; *p* = 0.0574; pretreatment (control and RB) F(1,35) = 1.46; *p* = 0.2360; groups x pretreatment F(1,35) = 1.54; *p* = 0.2226) (Figure 4). 

### 2.5. Effect of the Administration of Se and Tianeptine (TIA) in the FST 

**Figure 5:**
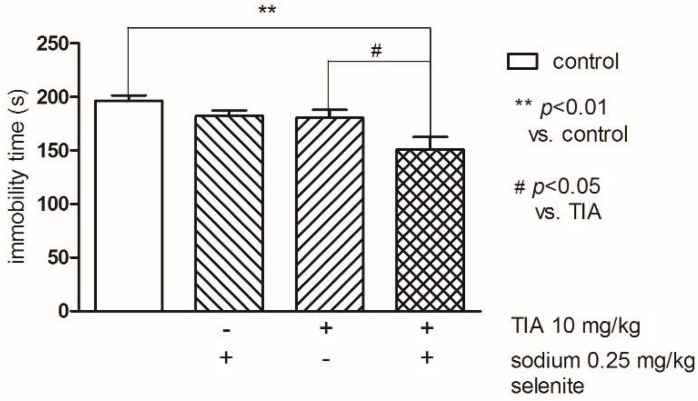
The effects of combined administration of Se and tianeptine (TIA) on the total duration of immobility in the FST in mice. The values represent the mean ± SEM. Sodium selenite was injected IP 30 min before the test. ** *p* < 0.001 vs. control vehicle-treated group, # *p* < 0.05 vs. TIA (Bonferroni’s test).

Two-way ANOVA indicated statistically significant differences between groups (control and sodium selenite) (F(1,31) = 7.04; *p* < 0.05). The post-hoc Bonferroni’s test showed that both, Se (0.25 mg/kg) and TIA at the threshold dose 10 mg/kg administered alone had no effect on the immobility time. Whereas concomitant treatment of Se and TIA at the abovementioned doses resulted in a statistically significant reduction of the immobility time compared to the control (*p* < 0.01) as well as to TIA (*p* < 0.05) groups (Figure 5).

### 2.6. Effect of Pretreatment with p-Chlorophenylalanine (pCPA) on the Antidepressant-Like Effect of Sodium Selenite in the FST and on Locomotor Activity of Mice

**Figure 6:**
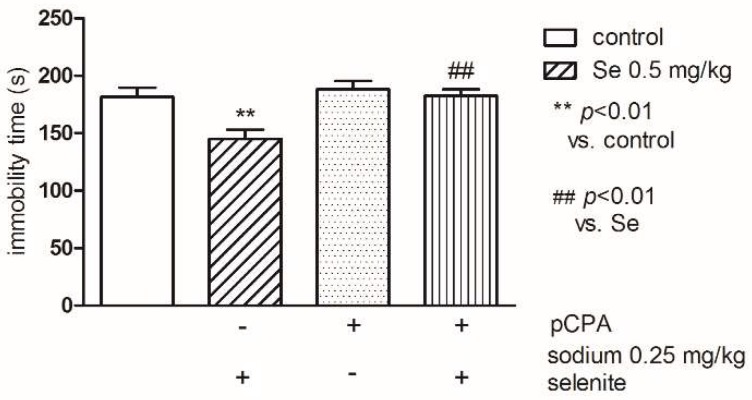
The effects of pretreatment with p-chlorophenylalanine (pCPA) on the antidepressant-like effect of sodium selenite in the FST in mice. The values represent the mean ± SEM. pCPA was injected at dose of 100 mg/kg for four consecutive days. Sodium selenite was injected IP 30 min before the test. ** *p* < 0.001 vs. control vehicle-treated group, ## *p* < 0.01 vs. pCPA (Bonferroni’s test).

Statistical analysis (two-way ANOVA) indicated statistically significant differences between groups (control and Se): (F(1,31) = 7.89; *p* < 0.01) and pretreatment (control and pCPA): (F(1,31) = 8.49; *p* < 0.01). Our results showed that the inhibitor of serotonin synthesis, pCPA alone (100 mg/kg, once a day, for four consecutive days) did not modify the immobility time, while pretreatment of mice with pCPA, significantly prevented the reduction of immobility elicited by sodium selenite, *p* < 0.01 (Figure 6).

### 2.7. Effect of the Administration of Se, Antidepressants, and pCPA on Locomotor Activity in Mice

Table 1 presents the effects of concomitant administration of Se with respective antidepressants and pCPA on spontaneous locomotor activity in mice. Two-way ANOVA did not reveal statistically significant differences; none of the tested drugs, either alone, at the used dose, or in combination with Se, had a statistically significant effect on the locomotor activity of mice.

### 2.8. Effect of the Administration of Se in the EPM

**Figure 7:**
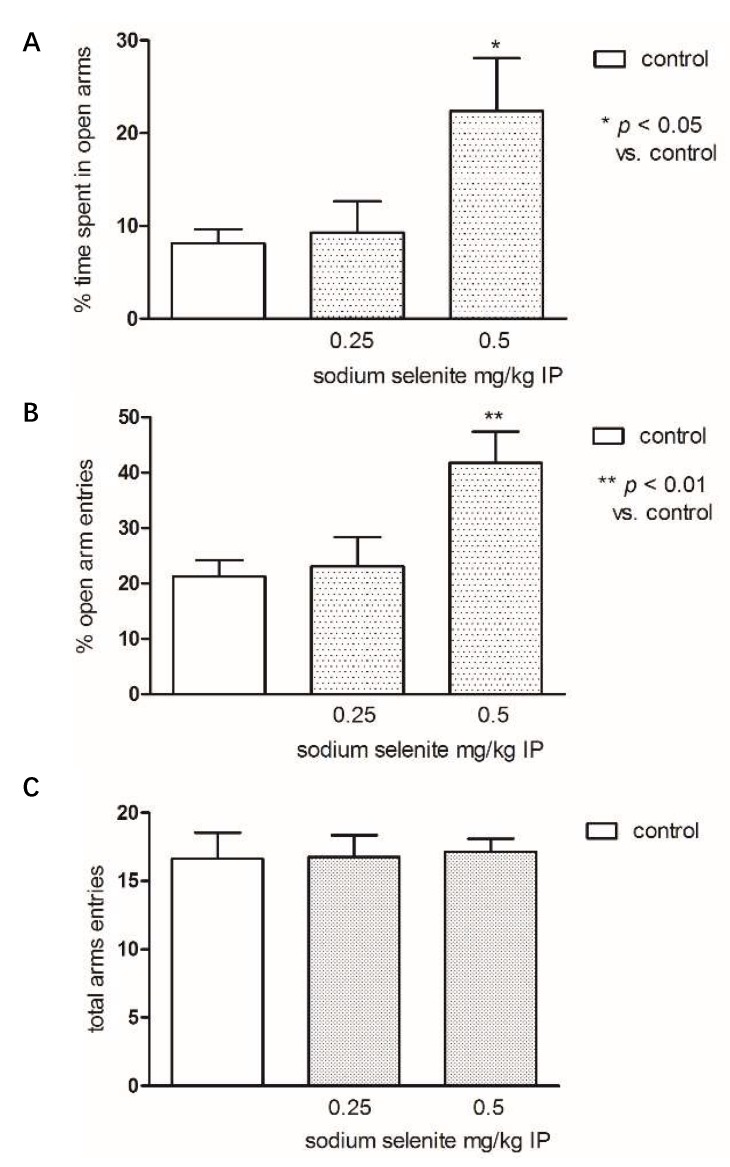
The effects of sodium selenite (at the doses 0.25 and 0.5 mg/kg) on the percentage of time spent in open arms: (**A**) percentage of open arm entries and (**B**) total number of arm entries (**C**) in the elevated plus-maze (EPM) procedure. Sodium selenite was administered IP 30 min before the test. Data are expressed as mean ± SEM values. ** *p* < 0.01; * *p* < 0.05 vs. control vehicle-treated group (Bonferroni’s test).

Figure 7 presents the effects of administration of Se in the elevated plus-maze (EPM). To determine the anxiolytic effect, Se was used in the following doses: 0.25 and 0.5 mg/kg. Selenium at a dose of 0.5 mg/kg significantly increased the percentage of time spent in the open arms compared to control group (one-way ANOVA: F(2,22) = 4.756; *p* < 0.05) as well as the percentage of open arm entries (one-way ANOVA: F(2,24) = 6.079; *p* < 0.01). There were no significant differences in the total number of entries into both arm types among the groups studied (one-way ANOVA: F(2,21) = 0.02842; *p* = 0.9720). Considering this result, the ineffective dose 0.25 mg/kg of Se was chosen for further interaction studies with diazepam in the EPM.

### 2.9. Effect of the Combined Administration of Se and Diazepam (DZ) in the EPM

**Figure 8:**
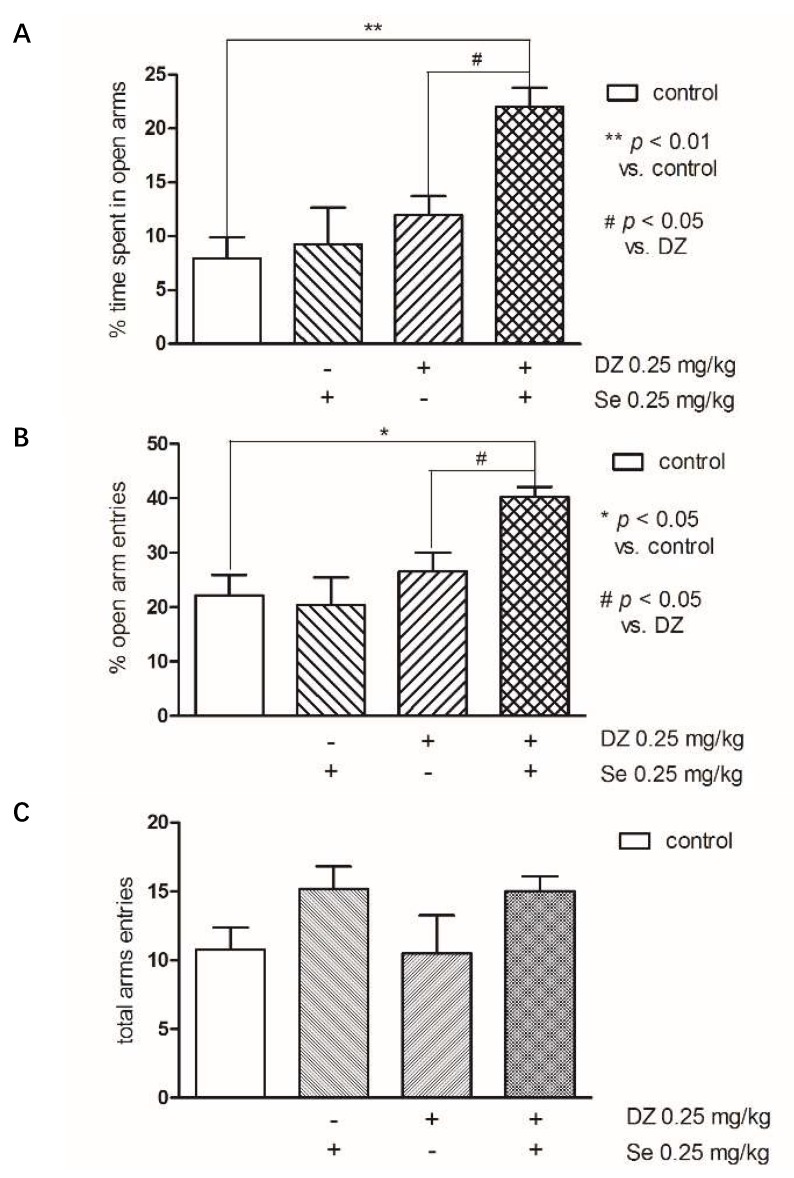
The effects of combined administration of Se and diazepam (DZ) on the percentage of time spent in open arms (**A**), percentage of open arm entries (**B**), and total number of arm entries (**C**) in an EPM procedure. DZ was injected subcutaneously (SC) 60 min and sodium selenite IP 30 min before the test. Data are expressed as mean ± SEM values. ** *p* < 0.01; * *p* < 0.05 vs. control vehicle-treated group, # *p* < 0.05 vs. DZ group (Bonferroni’s test).

Figure 8 presents the effects of combined administration of Se and diazepam (DZ) in the EPM. Combined administration of Se (0.25 mg/kg) and DZ (0.25 mg/kg) significantly increased a percentage of time spent in the open arms compared to the control group (*p* < 0.05), as well as to the DZ group (*p* < 0.05) (two-way ANOVA: groups (control and Se) F(1,24) = 4.83; *p* < 0.05; pre-treatment (control and DZ) F(1,24) = 10.69; *p* < 0.01). The percentage of open arms entries were also significantly increased compared to control group (*p* < 0.05), as well as to DZ group (*p* < 0.05) (two-way ANOVA: pre-treatment (control and DZ) F(1,21) = 9.79; *p* < 0.01). There was no significant impact of this treatment on the sum of all entries.

## 3. Discussion

Most of the experiments associated with depression and anxiety are focused around the monoamine theory, but there are studies pointing to other systems and mechanisms which may be involved in the pathogenesis of these disorders [32]. The presented study investigated the effect of Se (in the form of inorganic sodium selenite) on the action of antidepressants and anxiolytic diazepam, and also tried to determine the mechanisms responsible for the activity of this element. According to literature data, sodium selenite—present in many dietary supplements—is one of the most recommended compounds containing Se, especially in cancer chemotherapy [19,33] or in autoimmune disorders [34]. 

In the present study, sodium selenite was tested in the FST and EPM tests, which are well-known behavioral procedures [35,36,37]. The FST is widely used to evaluate the antidepressant activity of new drugs because it is sensitive to all major classes of antidepressants and is an important tool to study the neurobiological mechanisms involved in the antidepressant response. The EPM is the most commonly used test for studying the activity of anxiolytic or anxiogenic drugs [31,35,36,37,38]. 

The first stage of experiment, consisted of confirming the antidepressant- and anxiolytic-like potential of Se itself, as well as assessing the impact of this microelement on the locomotor activity of mice. Sodium selenite was used at an effective dose of 0.5 mg/kg, according to earlier studies [31], and a half smaller dose. The results obtained in this part allowed to determine the ineffective dose, 0.25 mg/kg, which was used in further research. The main objective of this study was to investigate the effect of Se, used in the form of inorganic sodium selenite, on the activity of antidepressants from various classes and anxiolytic diazepam, in FST and EPM tests in mice. An ineffective dose of sodium selenite—0.25 mg/kg in combination with threshold doses of selected drugs—was used in the experiments. Then, the pretreatment with p-CPA was made to check if the antidepressant-like effects of the sodium selenite would occur after inhibition of serotonin synthesis.

Imipramine is a tricyclic antidepressant (TCA) whose mechanism of action is based on the inhibition of competitive monoamine reuptake. It causes suppression of noradrenaline (NA) and serotonin (5-HT) uptake at a comparable level, while it has less influence on dopamine (DA) uptake. In addition to eliminating the main symptoms of depression, it also has anticholinergic effects, by blocking muscarinic receptors. It also acts as an antagonist of histamine, serotonergic, and adrenergic receptors, which has no effect on antidepressant efficacy, but only intensifies side effects [39]. Co-administration of Se and imipramine at ineffective doses caused a decrease in mouse immobility time in FST in comparison to the control group. This effect was not related to the increase in locomotor activity of rodents (Table 1).

Fluoxetine is the most commonly used antidepressant in the world. It belongs to a group of selective serotonin reuptake inhibitors (SSRIs), selectively inhibiting 5-HT reuptake. It is almost completely devoid of receptor action, making it safer to use [30]. This experiment showed that Se administered in an ineffective dose increased the effect of fluoxetine, also used at the threshold dose. The shortening of the animals’ immobility time in the FST was not correlated with the increase of their spontaneous motility.

Based on the above experiments, conducted with the inherence of imipramine and fluoxetine, it can be predicted that the shorter period of immobility of animals is due to the action of Se on 5-HT transmission. This hypothesis is consistent with the research of many authors. Brüning and co-authors [40] have demonstrated that the serotonergic (5-HT_1A_, 5-HT_2A/2C_ i 5-HT_3_) receptors and the opioid system are involved in the antidepressant activity of m-trifluoromethyl-diphenyl diselenide (m-CF_3_-PhSe)_2_. Similarly, a study by Gay et al. [41] confirmed the role of serotonergic transmission in the antidepressant activity of 3-(4-fluorophenylselenyl)-2,5-diphenylselenophen (DPS). Simultaneous administration of DPS and paroxetine in ineffective doses resulted in shortening immobility in FST. In addition, it was also shown that a single dose of DPS caused a significant inhibition of 5-HT re-uptake, suggesting an effect similar to SSRIs. Interaction with 5-HT transporters increased the level of 5-HT in synapses and led to activation of all serotonergic receptors in mice brains. This assumption could explain the synergism that occurs after combined administration of Se in ineffective dose with antidepressants. Analogous conclusions are also presented by Savegnago et al. [42] who described the participation of the serotonergic system in antidepressant activity of diphenyl diselenide (PhSe)_2_ in rats.

In our study, to confirm or exclude the contribution of the serotonergic system in the antidepressant-like activity of sodium selenite, we conducted an experiment with pCPA. Data already reported in the literature have shown that the administration of pCPA (an inhibitor of serotonin synthesis that blocks tryptophan hydroxylase) by four consecutive days depletes the endogenous stores of 5-HT by about 60% in mice, without influence on noradrenaline and dopamine levels [43]. P-chlorophenylalanine (pCPA) was reported to block the antidepressant-like effect of selective 5-HT reuptake inhibitors (SSRIs, such as fluoxetine and citalopram) in the TST and FST but not noradrenaline reuptake inhibitors (NRIs, such as reboxetine) or tricyclics (such as desipramine) [44,45]. This is consistent with the hypothesis that SSRI compounds elicit their acute behavioral effects by increasing extracellular 5-HT. In our experiments, pCPA alone did not affect the immobility time of mice in the FST in accordance with other reports [46,47], but reduction in brain 5-HT induced by the pCPA, prevented the antidepressant-like effect of sodium selenite, indicating an important role of this monoamine in its antidepressant-like effects in FST. 

Reboxetine is characterized by a specific mechanism of action. It is a selective NA reuptake inhibitor, and as a consequence, it does not affect serotonergic and dopaminergic transmission, which is associated with a reduction of side effects [48]. The experiment did not show any effect of Se at a dose of 0.25 mg/kg on the activity of reboxetine given in the ineffective dose (2.5 mg/kg). On this basis, it can be assumed that Se does not affect noradrenergic transmission. This assumption departs from some scientific reports. Posser et al. [27] showed that prazosin (α1-adrenergic receptor antagonist) and yohimbine (α2-adrenoceptor antagonist) were able to reverse the antidepressant effect of ebselen (C_13_H_9_NOSe), which may indicate the interaction of Se compounds with these receptors. However, the authors of the same publication, exclude the participation of serotonergic transmission.

Tianeptine belongs to the newest atypical antidepressants. Its mechanism of action is not fully understood. Despite its similar structure to TCAs, it is characterized by different pharmacological properties. The monoamine concept does not explain the efficacy of tianeptine. The ability to restore normal neuroplasticity to damaged areas of the brain by reversing changes in glutamatergic transmission caused by oxidative stress is considered the most likely mechanism of its action [49]. The conducted tests show that the combined administration of Se and tianeptine at ineffective doses shortens the period of immobility of mice in the FST. The obtained synergy may lead to speculation that the mechanism of antidepressant-like action of Se is related to its antioxidant potential. Supplementation of this microelement increases the amount of substrates for the synthesis of selenoproteins, including glutathione peroxidase, which is responsible for protecting cells against oxidative stress [28].

In order to examine the effect of Se on the activity of anxiolytic drugs, diazepam or the representative benzo-1,4-diazepine (BDZ) receptor agonist was used. The drug is characterized by anxiolytic, sedative, hypnotic, anticonvulsive, and myorelaxant properties. Its mechanism of action is increasing the affinity of endogenic GABA to GABA_A_ receptors, which indirectly increases the frequency of opening of chloride channels [50]. Simultaneous administration of Se and diazepam at ineffective doses resulted in longer time spent in the open arms of EPM and an increased number of entries into these arms. The obtained results seem to confirm the participation of GABA-ergic transmission in the anxiolytic action of Se. This assumption is consistent with the research of other authors. Ghisleni et al. [51] reported anxiolytic activity of (PhSe)_2_ in the EPM test. Furthermore, they demonstrated that the use of a GABA_A_ receptor antagonist, bicuculline, abolished the effect of (PhSe)_2_, suggesting that GABA_A_ receptors may play a role in the mechanism of action of this compound in the CNS. In addition, the same publication showed that the use of serotonergic receptor antagonists ritanserin, ketanserin, and WAY 100635 also resulted in the abolition of the anxiolytic activity of the tested compound, which confirms the previous assumptions regarding the contribution of 5-HT transmission. 

The obtained results clearly indicate that inorganic forms of Se possess antidepressant- and anxiolytic-like activity, which is consistent with literature reports referring to organic forms. Savegnago and co-authors [52] demonstrated antidepressant and anxiolytic activity of (PhSe)_2_ administered intragastrically in the FST test and in the tail suspension test (TST), without affecting animal motility. Such activity was attributed, at least in part, to the effects of Se on the pathway of nitric oxide (NO), associated with L-arginine and cyclic guanosine monophosphate (cGMP). Similar results were also presented by Oliveira et al. [39], where activity of the tested compound (CH3SePh) was based on the effect of Se on the dopaminergic system. We can speculate that the antidepressant-like effect of Se, obtained both in the present study and in the work of other authors [30], can be attributed to the rapid increase in the synthesis of selenoproteins, which are known to protect against lipid peroxidation and oxidative cell damage, or other mechanisms unrelated to selenoproteins [53]. Such explanations would be especially relevant in the context of association between low levels of cholesterol and increased risk of depression and suicidality proposed by De Berardis et al. [5,6,7]. According to this hypothesis, anti-lipoperoxidative effect of Se would contribute to its antidepressant effect [54]. Other mechanisms include: the effect of Se on calcium homeostasis [25], changes in phosphorylation of proteins or interactions with ion channels [55] and its anti-inflammatory properties [26]. Most often, however, Se could potentially elicit antidepressant- and anxiolytic-like effect through its modulatory role in various neurotransmitter systems. The hypothesis is proposed in which these effects of Se results from the influence on the 5-HT and GABA-ergic transmission and CNS functioning [28,29,30,31]. 

The limitation of the study is that one of the unique features of the FST is that it responds to acute treatment with antidepressant drugs and that these effects are augmented after chronic treatment [56]. Some antidepressants administered chronically produce their effects even at lower doses than those used in the FST [57]. Hence, chronic treatment with Se and investigation of its effects in animal models of depression would give better insight in its possible therapeutic value. Besides, it would be worth to use a more physiological route of Se administration (e.g., in feed), as well as to evaluate the effect of this supplementation on brain neurotrofin (especially BDNF) level. In addition, to confirm the participation of the 5-HT system, the affinity to receptors, transporters, and signal transduction pathways underlying the antidepressant-like effects of Se, further studies using various molecular techniques are needed.

## 4. Materials and Methods

### 4.1. Animals

The studies were conducted on 336 male Albino Swiss mice weighing 20–24 g. The mice were housed in cages, 8 individuals per cage. The ambient temperature was 22 ± 1 °C, standard lighting imitating day–night conditions was maintained. The animals were provided with free access to food (LSM, Motycz, Poland) and water, except for the short time that they were removed from their cages for testing. All experiments were carried out between 9:00 and 16:00, in accordance with the guidelines of Directive 2010/63/EU and accepted by the Local Ethics Committee (License No. 80/2016).

### 4.2. Drugs

Sodium selenite was purchased from Sigma–Aldrich and administered at doses of 0.25 and 0.5 mg/kg (equivalent to 0.112 and 0.225 elemental Se/kg body weight). Also, all antidepressants used were purchased from Sigma–Aldrich Company (St. Louis, MO, USA) and used at threshold doses, respectively: imipramine (IMI, 15 mg/kg), fluoxetine (FLX, 5 mg/kg), reboxetine (RB, 2.5 mg/kg), and tianeptine (TIA, 10 mg/kg). Diazepam (DZ, Relanium, Polfa, Poland) was used at threshold dose of 0.25 mg/kg. After a short period of animal adaptation to the room in which the experiment was conducted, the compounds were administered. Sodium selenite, dissolved in physiological saline, was injected intraperitoneally (IP), 30 min before the test. Antidepressants were administered IP, 60 min before the test, after the dissolution or dilution in physiological saline. Used doses and pretreatment times as well as systemic route of administration were selected on the basis of literature data and previous experiments from our laboratory, enabling a precise control of drug amounts [58,59,60]. To establish the involvement of the serotonergic mediated mechanism in the anti-immobility effect of sodium selenite (0.5 mg/kg) in FST, animals were pretreated with pCPA (p-chlorophenylalanine methyl ester hydrochloride) dissolved in saline, at a dose of 100 mg/kg once a day, for 4 consecutive days [47,61]. After the last pCPA injection, animals were treated with sodium selenite and tested in the FST 30 min later. Diazepam was administered subcutaneously (SC), and diluted in 0.9% saline containing 0.2% Tween 80. All compounds were injected in a manner generally accepted in experimental pharmacology, in an amount of 10 mL/kg body weight. Animals were weighed immediately before injection. Each study group consisted of 8–10 individuals. The control group received an equivalent volume of the physiological saline, in adequate time before testing. Between the injections, mice were provided with stable living conditions and unrestricted access to food and water.

### 4.3. Behavioral Tests

#### 4.3.1. Forced Swim Test (FST, Porsolt’s Test)

The study was carried out using the test proposed by R. Porsolt [35]. The method is based on the observation of an animal forced to swim in a situation where there is no possibility of escape. After the initial period of increased vigorous activity, the animal resigns from further attempts to escape. The test consists of immersing the mouse individually in a cylindrical vessel (diameter 10 cm, height 25 cm) filled with water at 23–25 °C up to a height of 10 cm, for a period of 6 min. Then, the time of immobility between the 2nd and 6th min is measured using stopwatches by an observer blinded to the treatment schedule. The immobility is considered to be the state in which the mouse performs only the movements necessary to keep the head above the surface of the water, adopting a semi-horizontal position. The immobilization of an animal is the equivalent of a human sense of hopelessness [62].

#### 4.3.2. Locomotor Activity

The spontaneous motility of mice was measured using a photocell apparatus (Multiserv, Lublin, Poland). The apparatus consisted of round cages (diameter 25 cm) made of plastics. The measuring element was made of infrared motion sensors. The results were presented on an liquid crystal display (LCD). Locomotor activity was interpreted as an interruption of light rays (motility count) by freely moving mice. The animals were placed in the cage individually, 50 min after the administration of antidepressants or diazepam, and 20 min after the injection of sodium selenite, for a period of 10 min for acclimatization. After this time, their activity was measured from the 2nd and 6th min, corresponding to the observation period in the FST.

#### 4.3.3. Elevated Plus Maze Test (EPM)

Anxiety behaviors were measured using the EPM test according to the Lister method [63]. The device was made up of four black-painted, crossed arms arranged in a plus sign and a central platform (5 × 5 cm). Two arms 30 × 5 cm were open, while the other two, 30 × 5 × 15 cm, were closed. Individual types of arms were placed opposite each other. The whole structure was elevated to a height of 45 cm above the floor. The observation lasted 5 min. The experiment was done in a quiet, dark room. The platform was lit with weak red, matte light. The mice were individually placed at the central square of the plus-maze apparatus, facing the open arm, while their behavior was observed for 5 min (using a stopwatch), by an observer blinded to the treatment schedule. The following measures were obtained from the test: the time spent in the open arms of the EPM, expressed as a percentage of the total exploration time, the number of entries into the open arms expressed as a percentage of the total number of entries into both types of arms. The total number of entries into either type of arm was used as a measure of overall locomotor activity. 

### 4.4. Statistical Analysis

The data obtained from the experiments were subjected to statistical evaluation. One- and two-way ANOVA analyses were used when appropriate and Bonferroni’s test was used as a post-hoc. The results are presented as the means ± SEM (standard error of the mean); *p* < 0.05 was considered as statistically significant. All analyses were prepared using the GraphPad Prism 5.0 program (San Diego, CA, USA).

## 5. Conclusions

The presented data generally confirm the antidepressant- and anxiolytic-like activity of Se and indicate its specificity, because the results obtained in the FST and EPM tests were not related to the increase in locomotor activity of the animals. In conclusion, Se, administered in the inorganic form of sodium selenite, may exert antidepressant- and anxiolytic-like effects hypothetically via endogenous selenoproteins or by affecting serotonergic and GABA-ergic neurotransmission. This preliminary evidence suggests that Se could be potentially valuable for the treatment of depression with coexisting anxiety, especially in polytherapy; however, to draw more accurate conclusions, more detailed preclinical as well as clinical studies are necessary.

## Figures and Tables

**Table 1:** Effect of the administration of sodium selenite Se (0.25 and 0.5 mg/kg), antidepressants, and p-chlorophenylalanine (pCPA) on locomotor activity in mice. The values represent the mean of the motility of mice between 2nd and 6th min ± SEM (standard error of the mean).

Treatment	Activity Counts	Treatment	Activity Counts
control	20.57 ± 3.538	Se 0.25 mg/kg	24.43 ± 4.364
Imipramine 15 mg/kg	21.44 ± 3.163	Imipramine + Se 0.25 mg/kg	15.89 ± 1.687
Fluoxetine 5 mg/kg	22.43 ± 6.55	Fluoxetine + Se 0.25 mg/kg	17.86 ± 4.458
Reboxetine 2.5 mg/kg	15.71 ± 1.629	Reboxetine + Se 0.25 mg/kg	18.43 ± 3.184
Tianepine 10 mg/kg	17.67 ± 4.359	Tianepine + Se 0.25 mg/kg	25.71 ± 7.187
pCPA 100 mg/kg	26.67 ± 4.842	Se 0.5 mg/kg	20.40 ± 2.015
		pCPA + Se 0.5 mg/kg	28.00 ± 2.76
